# Exploring the Role of Urocortin in Osteoporosis

**DOI:** 10.7759/cureus.38978

**Published:** 2023-05-13

**Authors:** Omar M Ismail, Omar M El-Omar, Umar N Said

**Affiliations:** 1 Faculty of Biology, Medicine and Health, University of Manchester, Manchester, GBR

**Keywords:** urocortin 1, treatment choices, osteoclast, osteoporosis, urocortin

## Abstract

Osteoporosis is a debilitating disease that affects over 200 million people worldwide. Overactive osteoclast activity leads to micro-architectural defects and low bone mass. This culminates in fragility fractures, such as femoral neck fractures. Treatments currently available either are not completely effective or have considerable side effects; thus, there is a need for more effective treatments. The urocortin (Ucn) family, composed of urocortin 1 (Ucn1), urocortin 2 (Ucn2), urocortin 3 (Ucn3), corticotropin-releasing factor (CRF) and corticotropin-releasing factor-binding protein (CRF-BP), exerts a wide range of effects throughout the body. Ucn1 has been shown to inhibit murine osteoclast activity. This review article will aim to bridge the gap between existing knowledge of Ucn and whether it can affect human osteoclasts.

## Introduction and background

The term ‘osteoporosis’ is derived from the Greek words ‘oustoun’ and ‘poros’, meaning porous bone. Osteoporosis is defined as ‘a disease characterised by low bone mass and micro-architectural deterioration of bone tissue, leading to enhanced bone fragility and an increase in fracture risk’ [1]. The World Health Organization (WHO) further defines osteoporosis as a bone density of 2.5 standard deviations or lower (T score of ≤-2.5) below the young healthy adult mean value (taken as a healthy 30-year-old adult) [1].

Osteoporosis is considered to be a ‘silent’ disease, as micro-architectural fractures occur over time, leading up to the symptomatic fracture. Therefore, the prevalence of osteoporosis is much higher than public opinion would deem. A condition such as breast cancer attracts much more attention than osteoporosis, even though the risk of developing the former in white females is one in nine and the risk of a hip fracture in the same population group is one in six [2]. There is also a significant socio-economic burden of osteoporosis; in 2010, the cost of osteoporosis to the United Kingdom was £4,725 million, and 158,726 quality-adjusted life years (QALYs) were lost [3].

Bone turnover is a lifelong and uninterrupted process, and it is thought that it takes 10-25 years to replace all old bone with new bone in humans [4]. Although the homeostatic mechanisms involved in bone remodelling are extremely regulated, imbalances in the system can occur, resulting in conditions such as osteopetrosis, osteopenia or, more critically, osteoporosis [5]. Insufficient peak bone mass, excessive resorption of the bone and insufficient formation of new bone all play a role in the pathogenesis of osteoporosis [6].

Although there are currently measures to treat and reduce fractures and a variety of treatments that can limit the progression of osteoporosis, they do not tackle the fundamental problem. Moreover, the various treatment options are not optimal and are accompanied by a large range of side effects, such as osteonecrosis of the jaw [1]. The large impact of this disease will only worsen due to increased human life span; thus, there is definitely a large unmet need for an ideal treatment that would be welcomed by millions of sufferers and the medical community. This review will explore the role of an anti-resorptive molecule, urocortin (Ucn), which may provide a solution.

Epidemiology

**Figure 1:**
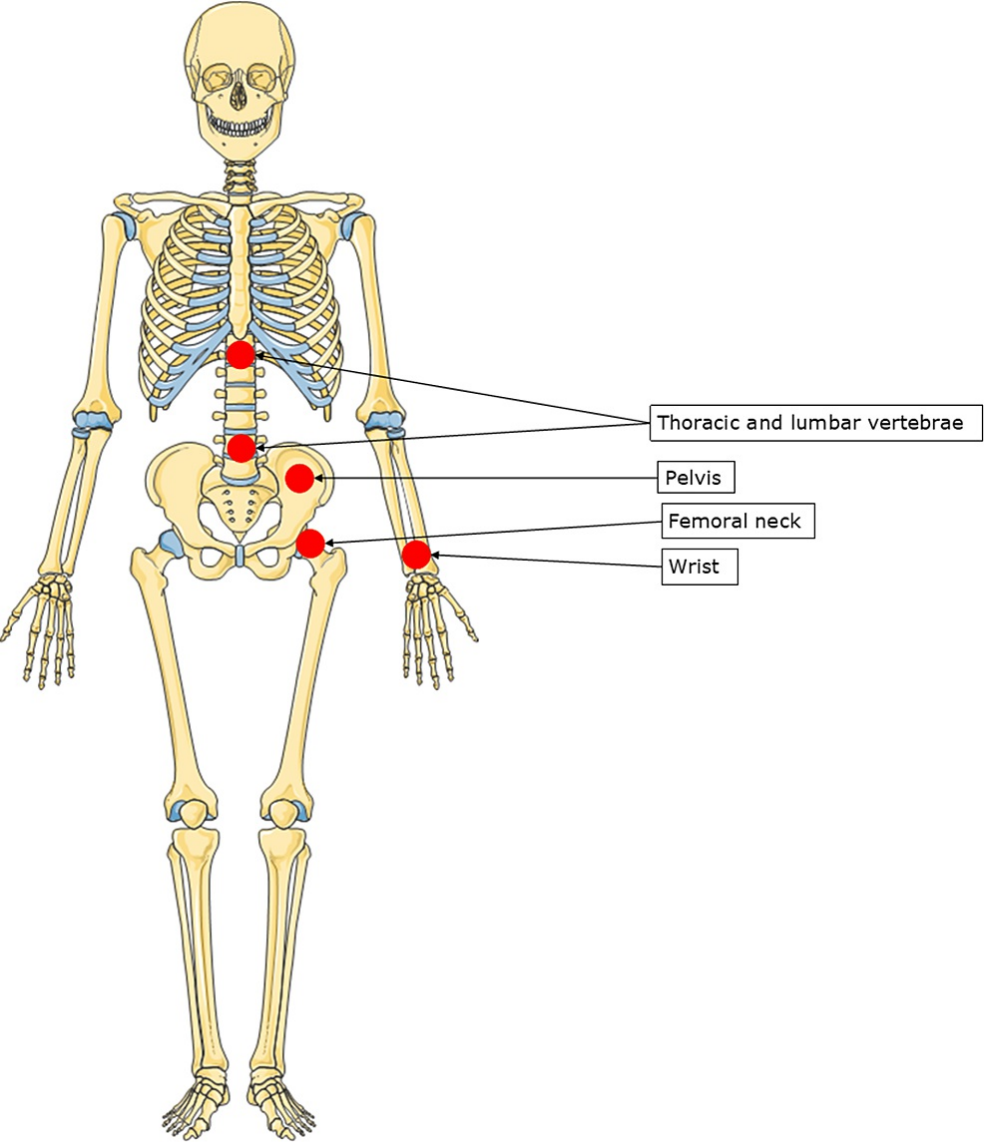
Most frequent locations where osteoporotic fractures occur. Circles highlight the locations of the most susceptible bones. Darker circles indicate higher economic cost to the state (in descending order: the femoral neck, vertebra, forearm and pelvis). Adapted from Hernlund et al. [3] and Skeletons and Bones [12] with permission

Two hundred million people are estimated to have osteoporosis worldwide, and thus, it is considered to be one of the more serious public health concerns globally [7]. Specifically in the United Kingdom, it is estimated that the prevalence of osteoporosis is over three million people, and 300,000 fragility fractures occur each year [8,9]. Expressed as a proportion, approximately 6% of males and 18% of females aged over 50 in the developed world suffer from osteoporosis [10]. Moreover, it is estimated that at the age of 50, one in two females and one in five males will eventually experience an osteoporotic fracture in the United Kingdom [2,11]. Osteoporosis becomes more common with age, and due to changes in lifestyle and ageing populations, the incidence and prevalence of osteoporosis are deemed to increase significantly in the future. It is estimated that in 2025, the cost of osteoporosis to the United Kingdom, including QALYs lost, will be £14,834 million, a 20% increase from the cost in 2010 [3]. The most frequent osteoporotic fractures and their respective, relative cost to the United Kingdom are highlighted in Figure 1.

Osteoporosis has an extremely profound impact on the NHS. Every year, hip fractures account for approximately 69,000 unplanned hospital admissions in England, 1.3 million bed days in English hospitals and £1.5 billion in English hospital costs alone, which does not account for the cost of community care [9]. Females who have experienced hip fractures also have a 10%-20% higher mortality than those without, demonstrating the serious morbidity and mortality associated with hip fractures [13].

## Review

Bone structure and physiology

**Figure 2:**
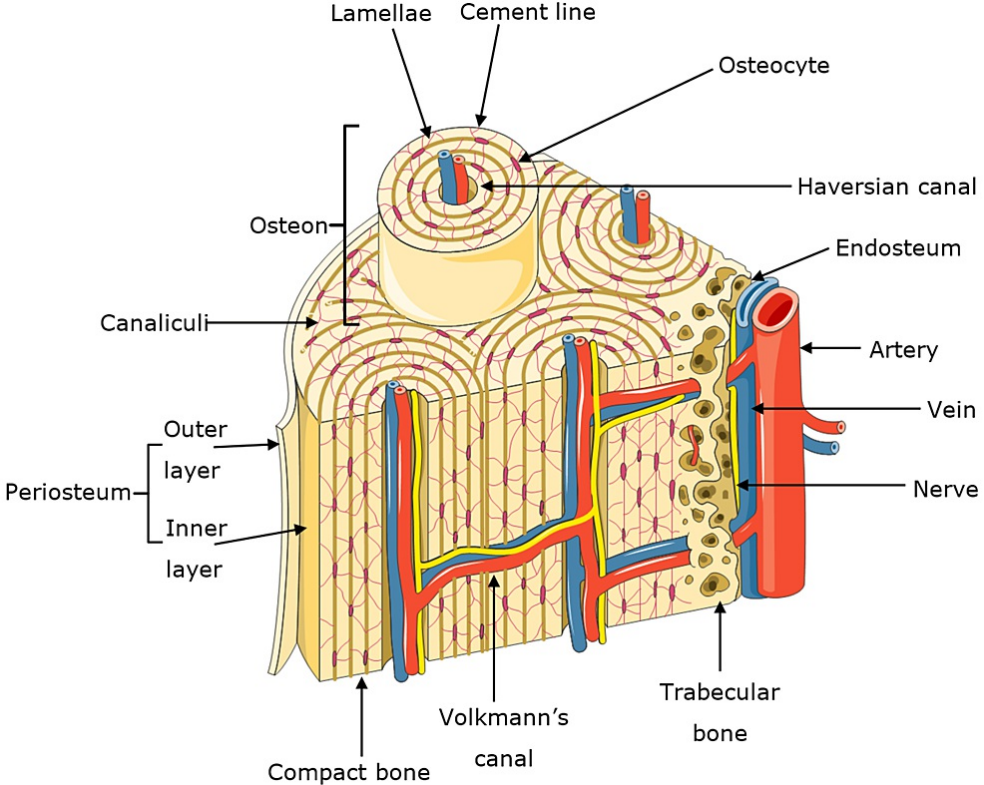
Cross-section of the bone. Overview of the cross-section of the bone, adapted from Skeletons and Bones [12] and Clarke [17] with permission

The bone is a mineralised connective tissue comprising different classes of cells including osteocytes, osteoblasts and osteoclasts [14]. Another constituent of the bone is an inorganic material, predominantly composed of phosphate and calcium ions [15]. These ions nucleate to form hydroxyapatite crystals [14]. Non-collagenous matrix proteins, with collagen (mostly composed of type I collagen), form a scaffold where hydroxyapatite can be deposited, and this scaffold gives the bone its stiff and structurally resistant character [16]. An overview of a bone cross-section is evident in Figure 2.

Bone cells

Osteocytes

Osteocytes make up 90%-95% of the total bone cells and are the cells with the longest life span, up to 25 years [18]. They are located within lacunae, embedded in a bone matrix, with varying morphology depending on the bone type in which they are in [19]. For example, more rounded osteocytes can be found in trabecular bone, compared to elongated ones found in cortical bone [20].

Osteocytes are derived from osteoblasts in the mesenchymal stem cell (MSC) lineage. At the end of each bone formation cycle, a group of osteoblasts differentiate into osteocytes, which are deposited into the bone matrix [14]. Various ultrastructural changes occur, such as a reduction of the round osteoblast size, a decrease in the number of organelles including the Golgi apparatus and rough endoplasmic reticulum and an increase in the nucleus-to-cytoplasm ratio. This equates to a decrease in the synthesis and secretion of protein [21].

When the osteocyte is fully implanted in the mineralised bone matrix, previously expressed osteoblast markers (including osteocalcin {OCN}, bone sialoprotein II {BSPII}, collagen type 1 and alkaline phosphatase {ALP}) are downregulated, and there is an increase in osteocyte marker expression, such as dentin matrix protein 1 (DMP1) and sclerostin [22-24].

Cytoplasmic processes for each osteocyte cell pass through miniscule tunnels known as canaliculi, originating from the lacuna space. This results in the osteocyte lacunocanalicular system [25]. The osteocytes connect with each other and the bone surface via cellular processes, such as gap junctions [17]. These gap junctions are composed of, and maintained by, connexion proteins. These gap junctions are necessary for the activity and survival of osteocytes [26].

Osteocytes act as mechanosensors, and their network can detect mechanical loads and pressures, aiding the bone to respond and adapt to daily mechanical forces [19]. The stress signals from various shear forces are transduced into biological activities by the osteocytes. Responses in osteocytes also result from the flow of canalicular fluid, influenced by external forces. Various signalling mechanisms utilised upon mechanical stimulation involve prostaglandin E2, cyclooxygenase-2 (COX-2), various kinases and nitric oxide (NO) [17]. Osteocytes may also undergo apoptosis in response to mechanical strain or oestrogen deficiency and glucocorticoids, which can result in osteoporosis [1,17].

Osteoblasts

Osteoblasts are produced from stimulated osteoprogenitor cells, which arise from pluripotent MSCs, and are known for their bone-forming abilities, secreting osteoid on the bone matrix [1,17,27]. Mature osteoblasts express the integrins α2β1 and ανβ1, indicating that they have the capability of secreting extracellular matrix (ECM) and forming new bone [17,28,29]. Osteoblasts also have receptors that bind ECM proteins, growth factors, cytokines and regulatory hormones (including parathyroid hormone {PTH}, vitamin D, leptin and oestrogen). Moreover, they can express numerous factors that regulate osteoclast differentiation and function [30]. For instance, they produce receptor activator of nuclear factor kappa-B ligand (RANKL), which binds to receptor activator of nuclear factor kappa (RANK) receptors on the osteoclast, triggering osteoclast maturation and bone resorption [30,31]. Osteoblasts will eventually become ingrained in the ECM, indicating the initial phase of apoptosis where they transform into osteocytes [17].

Osteoclasts

Activated multinucleated osteoclasts are derived from the same haematopoietic progenitor cells that also differentiate into monocytes and macrophages. They form upon the fusion of mononuclear precursors and have a limited life span of only approximately two weeks [30]. They are the only cells capable of bone resorption [17]. Macrophage colony-stimulating factor (M-CSF), RANKL, interleukin 1 (IL-1) and tumour necrosis factor (TNF) are some of the important cytokines and growth factors that regulate human osteoclast formation [17,30]. M-CSF in particular is required for osteoclast precursor differentiation, proliferation and survival [17]. Osteoclast maturation and differentiation occur via stromal cells or osteoblasts expressing RANKL, which binds to RANK receptors localised on the osteoclast precursor surface. M-CSF stimulates precursor cells to differentiate into osteoclasts. Osteoprotegerin (OPG), secreted by stromal cells, can prevent RANKL binding to RANK and thus is a regulator of osteoclast differentiation [30].

Bone resorption

RANKL is integral to starting the resorptive process. When RANKL binds with RANK, there are a number of structural changes in the osteoclast. For example, the osteoclast body becomes polarised and forms a cytoskeleton that is rich in filamentous actin (F-actin), and a dense podosome belt is also formed that surrounds the osteoclast [31,32]. Podosomes are conical, actin-rich structures that protrude from the osteoclast plasma membrane and attach to the bone matrix; the podosome belt then forms a thick actin ring during resorption [33]. The podosome ring, along with the ανβ3 integrin, creates a sealing zone between the osteoclast and the bone matrix, allowing the two to adhere to each other. This sealing zone is important to ensure efficient bone resorption via an acidified surrounding (pH of 4.5) with a high protease concentration [34].

**Figure 3:**
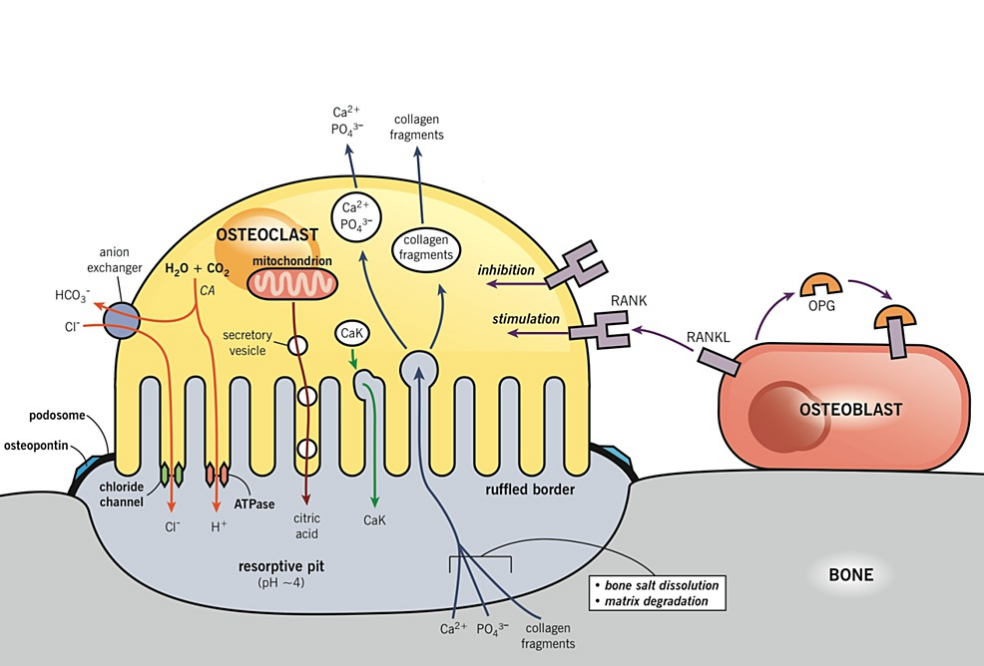
Summary of the mechanism of bone resorption. RANKL is expressed by osteoblasts, which binds to RANK receptors expressed by the osteoclast, stimulating formation and function as described earlier. The villous projections of the osteoclast form a ruffled border around the bone area to be absorbed. This is aided by podosomes and osteopontin (an associated link protein), which form an adhesive ring around the ruffled border. The result is an isolated resorptive pit. CO_2_ and H_2_O form carbonic acid, which dissociates into an H+ cation and a bicarbonate ion (HCO_3_-). The H+ is substituted for a chloride ion (Cl-). H+ and Cl- form hydrochloric acid, which breaks down the bone. Furthermore, lysosomal enzymes are also secreted by the osteoclast to further break down the bone. The resorptive pit is shielded from extracellular fluid and other environmental stimuli, to ensure an acidic, resorptive environment. Taken from Asagiri and Takayanagi [35] and Calcium homeostasis and osteoporosis [36] with permission RANKL, receptor activator of nuclear factor kappa-B ligand; RANK, receptor activator of nuclear factor kappa; OPG, osteoprotegerin

The secretion of hydrochloric acid and cathepsin K enzyme by osteoclasts initiates bone resorption [17]. The resorption compartment beneath the osteoclasts is broken down by the acidic H+ ions, dissolving the mineralised component of the bone matrix; concurrently, the lytic enzymes digest the protein element of the matrix, mainly composed of type I collagen. Moreover, the bone matrix debris is endocytosed by the osteoclasts, processed and then released into the circulation [32]. A summary of this process is visualised in Figure 3.

RANKL/RANK/OPG pathway

RANKL, RANK and OPG are all part of the TNF superfamily of proteins, which oversee bone remodelling and thus bone mass and density [37]. RANKL is expressed in a membrane-bound form (more potent at commencing osteoclastogenesis) by osteoblasts and osteocytes, but following proteolytic cleavage by matrix metalloproteases (MMPs), RANKL can become soluble [38]. On the other hand, the RANK receptor is expressed by pre-osteoclasts and osteoclasts [32]. When RANKL binds to the RANK receptor, this subsequently results in osteoclastogenesis, bone resorption and H+ ion secretion [39].

Osteoprotegerin (OPG) is a soluble cytokine receptor, secreted by osteoblasts and osteocytes, that intercepts and binds strongly with RANKL to inhibit its action at the RANK receptor, stopping RANKL-RANK receptor complexes forming [22,40].

Osteoporosis

Aetiology

There are numerous interacting factors that may predispose one to a higher risk of osteoporotic fractures. Firstly, regarding primary (senile) osteoporosis, a low peak bone mass is a major indication of below-average bone mass density (BMD) in older individuals, which is associated with an increased risk of fracture [41,42].

Moreover, postmenopausal females with a low percentage of body fat or a low body mass index are also at an increased risk of abnormal bone loss and low bone mass, both of which contribute to postmenopausal osteoporosis [43]. In addition, menopause is associated with lower oestrogen levels, and low serum total oestradiol concentrations (<5 pg/mL) can increase the risk of hip and vertebral fractures, regardless of the BMD [44].

A previous fracture, particularly in females, can lead to an increase in the likelihood of a new vertebral fracture in subsequent years; one in five females will have a fracture in the following year [45]. Poor hand grip strength and impaired vision together also contribute to the prospect of falls, which is a major independent risk factor for fractures [46,47].

There are also various medical disorders that can lead to secondary osteoporosis, such as gastrointestinal conditions (malabsorption diseases that lead to reduced calcium ion uptake) and haematological diseases (e.g. pernicious anaemia) [48]. Certain medications can also exacerbate or predispose one to osteoporosis. Glucocorticoids in particular have been shown to affect the quality and volume of the bone, which is problematic for elderly patients with rheumatoid arthritis [49].

Finally, vitamin D deficiency can result from a lack of exposure to sunlight and a lack of calcium intake. In these instances, due to the high bone turnover and secondary hyperparathyroidism leading to the loss of bone mass, the risk of fracture is thus much higher [50].

Pathogenesis

**Figure 4:**
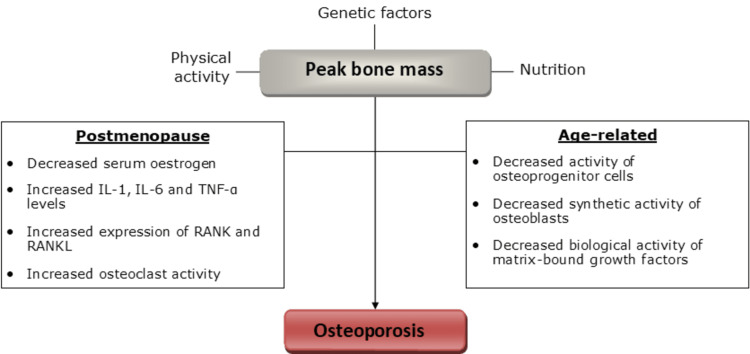
A summary of the pathophysiology in osteoporosis. Image credit: OM Ismail IL-1, interleukin 1; IL-6, interleukin 6; TNF-α, tumour necrosis factor-alpha; RANK, receptor activator of nuclear factor kappa

There are two main forms of osteoporosis: age-related (low-turnover variant) and postmenopausal osteoporosis (high-turnover variant). Calcium levels also perform a fundamental role. The summary of the pathophysiological processes can be seen in Figure 4.

There is evidence of a number of age-related changes in the bone matrix [30]. Osteoblasts, isolated from the blood of the elderly population, demonstrated a lower proliferative and biosynthetic potential in comparison to the osteoblasts from the younger population [51]. Moreover, growth factors (which stimulate osteoblast activity) that are bound to the ECM become less potent over time. Overall, there is a net loss in bone density [30].

Oestrogen plays an important role in bone remodelling. In postmenopausal osteoporosis, oestrogen deficiency stimulates blood monocytes and bone marrow cells to secrete greater levels of inflammatory cytokines. These stimulate osteoclast activity via boosting the levels of RANKL and reducing the expression of OPG. Although there is some compensatory bone formation by osteoblasts, it is overshadowed by the high rate of resorption [30].

Calcium is a necessary composite of the bone, continually replaced along with the bone. Regarding the calcium nutritional state, a trend of inadequate dietary calcium has been seen in adolescent females (but not in males). At this young age, there is rapid bone growth, and this point in time is where calcium reserves are built up for the remainder of adult life. Reduced calcium intake leads to stunted peak bone mass and thus a greater likelihood of osteoporosis in the future. Vitamin D deficiency, increased PTH concentrations and calcium deficiency all can contribute to the development of age-related osteoporosis [30].

Clinical Features

In the absence of complications, osteoporosis is usually asymptomatic until a fracture occurs. It would be characterised by a sudden onset of severe pain, exacerbated by any movement [1]. Typical osteoporotic fractures include those of the hip, vertebral body and distal forearm [52].

Investigations

Regarding acute fractures, after taking a detailed patient history, an X-ray will confirm the fracture and may also reveal previously asymptomatic vertebral deformities. Furthermore, to investigate bone densitometry, a dual-energy X-ray absorptiometry (DEXA) scan of the hip can be carried out. This is the ‘gold standard’ in osteoporosis diagnosis [1].

Current managements

Lifestyle

The National Institute for Health and Care Excellence (NICE) recommends various preventative measures and lifestyle changes that patients at risk of osteoporosis or who have osteoporosis can undertake. Particularly, smoking cessation and a reduction in alcohol consumption are beneficial, along with calcium- and vitamin D-rich diets. Outdoor exercise will increase exposure to sunlight and thus increase vitamin D production, whilst strength and weight training can increase bone mineral density [53]. Balance exercises, including Tai Chi, and occupational therapy home visits can also reduce the risk of falls, which would lead to fragility fractures [11,53].

Pharmacological Intervention

Patients who are at high-risk for osteoporotic fractures are offered pharmacological measures, which reduce that risk. Lifestyle advice and adequate dietary calcium intake would be taken in combination with these medications, outlined by NICE guidance. Each drug is prescribed contingent on age, the T score and the number of fracture risk factors. However, each medication also has adverse effects.

Bisphosphonates

Bisphosphonates are analogues of bone pyrophosphate [1]. Bisphosphonates bind to hydroxyapatite crystals in the bone matrix and inhibit farnesyl pyrophosphate synthase, an enzyme in the mevalonate pathway [1,54]. This pathway produces intermediates that are utilised to manufacture isoprenoids; this results in the prenylation of certain proteins such as Rho GTPases, allowing for the attachment of lipids to proteins. This step is crucial for cytoskeletal organisation in osteoclasts, so when bisphosphonates inhibit farnesyl pyrophosphate synthase, it subsequently disrupts cytoskeletal structure and osteoclast motility and resorption [53].

Alendronate and risedronate are most commonly prescribed in this class as once-weekly doses [1]. Alendronate can also be prescribed to patients who take glucocorticoids [55]. Although side effects are usually minimal, they can include upper gastrointestinal symptoms such as oesophagitis. To combat this, patients should remain standing or sitting upright and avoid food and drink for at least 30 minutes following ingestion (in a fasting state with large amounts of water), which can be restrictive. Moreover, the prolonged use of bisphosphonates can also lead to side effects that include atypical femoral fractures and osteonecrosis of the jaw, although the benefits often outweigh these adverse effects [1].

Denosumab

Denosumab is a monoclonal antibody to RANKL, administered as a subcutaneous injection every six months. Its anti-resorptive properties also inhibit osteoclast activity and are equivalent to bisphosphonates concerning fracture risk reduction ability [1]. This class of drugs is very useful in patients who cannot tolerate bisphosphonates, and it is administered every six months as a subcutaneous injection. However, it has been reported to increase the risk of cellulitis and skin rash [55].

Strontium Ranelate

A second alternative for those intolerant to bisphosphonates is strontium ranelate. Strontium is similar to calcium structurally and reduces reabsorption and promotes the formation of new bone [11]. It is not as potent as bisphosphonates and can give side effects including nausea, diarrhoea and headaches. There is also a small increased risk of venous thromboembolism (VTE) [1].

Raloxifene

Raloxifene is an oestrogen agonist/antagonist taken daily as a tablet. It is not usually recommended for patients to reduce the risk of invasive breast cancer in postmenopausal females with osteoporosis, as opposed to those solely with primary osteoporosis. It should be noted that there has been no evidence of reducing the risk of non-vertebral fracture, and side effects include leg cramps and flushing. VTE is also a complication that may occur with this intervention [55].

Hormone Replacement Therapy (HRT)

HRT can prevent osteoporosis in females with premature menopause or those with perimenopausal symptoms by augmenting the levels of oestrogen. Otherwise, there is an unfavourable risk-benefit ratio as it is associated with a wide range of adverse effects. The heightened risk of breast cancer and cardiovascular disease, including myocardial infarction, stroke and VTE, means that this treatment must always be carefully considered. When stopping HRT, bone volume may decrease quite substantially, so other agents must be used to maintain BMD [55].

Teriparatide

Teriparatide is a recombinant parathyroid hormone that stimulates bone formation through its anabolic properties, delivered via a daily subcutaneous injection. It is often considered in patients with a very high risk of fractures and who cannot tolerate bisphosphonates. Side effects include leg cramps, nausea and dizziness. It is recommended that this regimen does not exceed 18-24 months, as there is evidence in rats that teriparatide can cause osteosarcoma in long-term usage. For the same reason, it is contraindicated in patients at risk of skeletal malignancy [55].

Urocortin system

Urocortin (Ucn) is a peptide that has been shown to have some potential use in rheumatoid arthritis and osteoarthritis, and it is hypothesised that it can be exploited for treating osteoporosis [56,57]. This system is made up of Ucn ligands, corticotropin-releasing factor (CRF) receptors and the ligand trap CRF-binding protein (CRF-BP). The Ucn ligands bind with the CRF receptors with various affinities, and diverse downstream signalling pathways may be activated [58]. The ligand trap CRF-BP can intercept any Ucn ligands and is thus a regulator of Ucn function [59].

Etymology and Structure

The urocortins (urocortin 1 {Ucn1}, urocortin 2 {Ucn2} and urocortin 3 {Ucn3}) are three paralogs of the peptide corticotropin-releasing factor (CRF), which is present in bony fish, amphibians, birds and mammals [60].

Urocortin 1

The first urocortin discovered, urocortin 1 (Ucn1), was termed so for its comparable primary structure and bioactivity to both urotensin 1 and CRF [61]. CRF and Ucn1 have similar affinities for CRF receptor 2 (CRF2); thus, the existence of more specific CRF2 ligands was suggested, which lead to the discovery of Ucn2 and Ucn3 [60-62].

The *Ucn1* gene codes for a 122-amino acid residue pre-protein, which is cleaved into an active form of 40 amino acids [60]. The human *Ucn1* gene is located on chromosome 2 (2p23-p21) possessing two exons, the second of which solely contains the coding region, similarly as the *CRF* gene [63]. Although not confirmed, it is assumed that in the *Ucn1* gene, some regulatory elements include a TATA box, a cyclic adenosine monophosphate (cAMP) responsive element (CRE), some GATA-binding sites, a cytosine-cytosine-adenosine-adenosine-thymidine (CCAAT) enhancer-binding protein (C/EBP) transcription factor-binding site, an oestrogen response element and Brn-2-binding sites [64,65].

Urocortins 2 and 3

Identified six years later in 2001, the prohormones urocortin 2 (Ucn2) and urocortin 3 (Ucn3) were perceived to have similar structure to CRF and Ucn1 pre-proteins. They were named ‘urocortins’ for their affinity for the CRF2 receptor [66,67]. Ucn2 and Ucn3 are considered equivalent to human stresscopin-related peptide and stresscopin, respectively [60].

The Ucn2 peptide is located in the C-terminus of a 112-amino acid residue pre-protein, whereas the Ucn3 peptide is located in a 161-residue pre-protein, both of which are cleaved into active forms of 38 amino acids [66,67]. The genes encoding Ucn2 and Ucn3 are situated on chromosomes 3 (3p21.3-4) and 10 (10p15.1), respectively [67,68].

**Figure 5:**
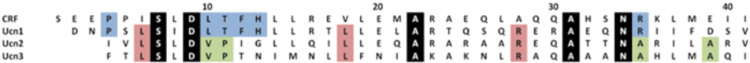
A comparison of the primary structure of the human Ucn/CRF family peptides. The boxed regions represent the amino acid sequence. The black fill represents CRF superfamily homology (five residues). The blue fill indicates selective type 1 Ucn1/CRF homology (six residues). The green fill indicates selective type 2 Ucn homology (four residues). The red fill indicates pan-Ucn homology (three residues). This demonstrates how CRF and Ucn1 are structurally similar and how Ucn2 and Ucn3 are structurally similar: ‘type 1 (Ucn1/CRF)’ versus ‘type 2 (Ucn2/Ucn3)’. Adapted from Fekete and Zorrilla [60] with permission Ucn, urocortin; Ucn1, urocortin 1; Ucn2, urocortin 2; Ucn3, urocortin 3; CRF, corticotropin-releasing factor

In terms of primary structure (amino acids), as highlighted in Figure 5, Ucn1 resembles CRF much more closely compared to how Ucn1 resembles Ucn2 or Ucn3. On the other hand, Ucn2 and Ucn3 also resemble each other closely. Considering these two Ucns are also more selective for the CRF2 receptor, these are considered to be ‘type 2 urocortins’ [69].

Pharmacology of Urocortin

**Figure 6:**
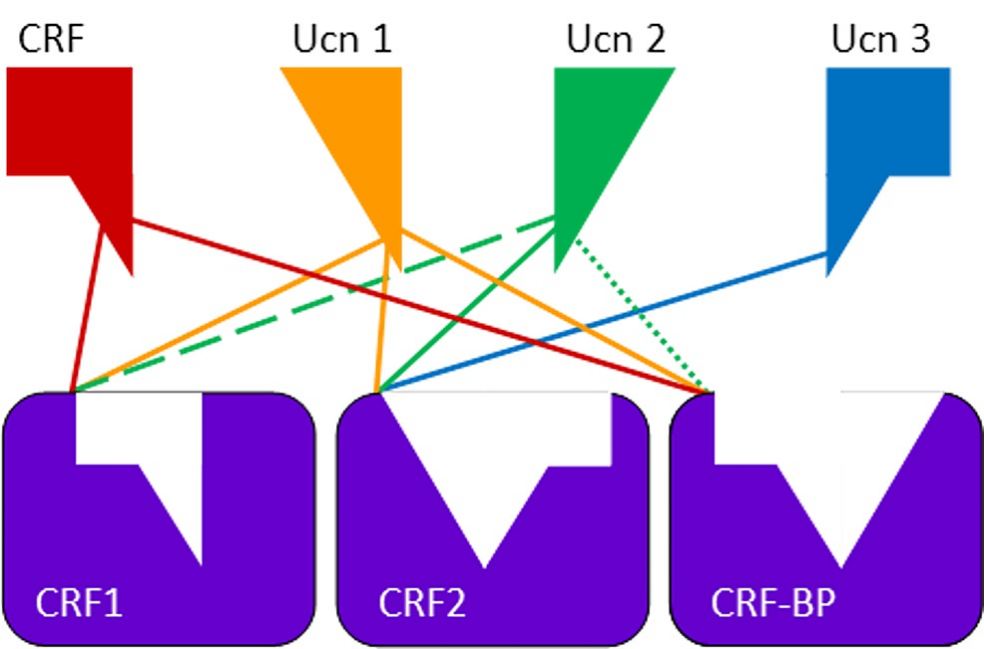
A summary of the affinity of CRF and each Ucn paralog to bind to each CRF-binding site. The dashed line represents a relatively low binding affinity. Adapted from Giardino and Ryabinin [75] with permission CRF, corticotropin-releasing factor; CRF1, CRF receptor 1; CRF2, CRF receptor 2; CRF-BP, CRF-binding protein; Ucn1, urocortin 1; Ucn2, urocortin 2; Ucn3, urocortin 3

Ucn1 has a very high affinity for all known CRF-binding sites. These include the CRF1 and CRF2 receptor families and the CRF-binding protein (CRF-BP), all of which will be discussed further in this section [61,67,70]. On the other hand, the type 2 Ucns (Ucn2 and Ucn3) only demonstrate affinity to certain CRF2 receptors and a lack of any or only limited affinity to CRF-BP [66-68]. Furthermore, the CRF receptors are part of a G protein-coupled receptor (GPCR) family and are also known as seven-transmembrane receptors as they pass through the plasma membrane seven times [71]. For each receptor, there are also various splice variant isoforms, including membrane-bound and soluble isoforms [72-74]. When stimulated, these receptors secrete adrenocorticotropic hormone (ACTH) when stimulated. See Figure 6 for a summary of the affinity of each Ucn to bind to each CRF-binding site.

CRF1 Receptor

The CRF1 receptor is a GPCR in the secretin receptor family, specifically subfamily B1, spanning approximately 415 amino acids [76,77]. There are at least eight spliced alternative isoforms of CRF1 in humans, but only one of these isoforms, CRF1(α), is known to have the ability to induce signal transduction in humans and rats, which will be explored in this section [60,77,78].

Ucn1 has the ability to reversibly bind to the CRF1 receptor with high affinity and is approximately three times more potent than CRF binding to the CRF1 receptor. Moreover, Ucn1 is also 1-2.5 times more potent than CRF at stimulating the CRF1-expressing cells to generate cAMP [60]. Unlike Ucn1, the type 2 Ucns have much lower potencies to bind to CRF1 [67]. Ucn3 in particular shows no functional ability to activate ACTH release in vitro from CRF1 receptors; however, Ucn2 can act, albeit at low potency, as a full agonist at CRF1 receptors [79].

CRF2 Receptor

CRF2 receptors also belong to the GPCR subfamily B1, and there are four splice variants that have been analysed: membrane-bound and soluble isoforms of CRF2(α) receptor and membrane-bound isoforms of CRF2(β) and CRF2(γ) receptors [60,80]. The length of the amino acid chains of the membrane-bound isoforms is 411, 431 and 397, respectively [81,82]. The soluble variant of CRF2(α) has a length of 143 residues and has been identified in mice [72]. As opposed to CRF, in humans, all Ucns bind with high affinity to the membrane-bound CRF2 receptors; Ucn2 and Ucn3 are approximately 1-2 orders of magnitude more potent at binding to CRF2 receptors compared to CRF, with Ucn1 being only slightly less potent than Ucn2 and Ucn3 [60]. With regard to binding to the soluble CRF2(α) receptor, Ucn1 and CRF (considered to be CRF1 ligands) have a very high affinity, and Ucn2 and Ucn3 have an unusually low affinity [72].

Ucn2 and Ucn3, the type 2 Ucns, are much more selective CRF2 agonists compared to Ucn1, due to their high potency towards membrane-bound CRF2 receptors and their low potency to CRF1 receptors. In the case of Ucn3, there is no potency towards CRF1 receptors at all [60]. Thus, Ucn2 and Ucn3 bind more preferentially to CRF2, whereas CRF and Ucn1 bind more preferentially to CRF­1.

CRF-Binding Protein (CRF-BP)

CRF-BP is a secreted glycoprotein in human plasma that can bind with CRF with high affinity [83]. This modulator of CRF activity is made up of 322-amino acid residues, and it putatively inhibits agonists of the CRF receptors by gathering secreted CRF or Ucn and promoting their degradation by enzymes, reducing the amount of circulating peptides binding to the receptors [59]. However, it has also been observed that CRF-BP augments the effects of the bound ligand; this is achieved by protecting the ligand from metabolic degradation during diffusion to the membrane-bound CRF receptors [84].

In mammals, Ucn1 is approximately as potent as CRF in terms of binding to CRF-BP [60]. Ucn1 has a specific region for its affinity to the CRF-BP sites and a separate region for the CRF receptors [80,83,85]. The type 2 Ucns do not remarkably bind to the CRF-BP in humans [60,67].

Signalling Pathways

Although different Ucn peptides exhibit varying affinities for each of the CRF receptors, these Ucns also exhibit a high degree of receptor signalling promiscuity. This trait is explained by Ucns having the ability to couple to multiple G proteins. Different G proteins can even be utilised in the same cell. As a result, there is a diverse range of intracellular networks that can be activated, involving cAMP, intracellular ions, nitric oxide (NO) and a variety of protein kinases [58]. Receptors are specific to the tissues, and the downstream signalling pathways are also specific to the effect that follows their activations. The mitogen-activated protein kinase (MAPK) pathways are some of the common pathways that are activated by the Ucn ligands [86].

Distribution of urocortin

Central Nervous System (CNS)

In the brain, Ucn1 is predominantly synthesised and distributed in the caudal areas, specifically in the Edinger-Westphal nucleus (EW) [87]. The dorsal midbrain EW structure has been identified as an integral component in oculomotor and pupillary function; however, present Ucn1-expressing neurons in the EW are believed to have functions outside of the oculomotor remit. The reason for this is that these neurons are not preganglionic cholinergic neurons, unlike others in the EW [88]. Some of these non-oculomotor functions include the homeostasis of temperature and food and water intake, behavioural responses to stressors, motor and vestibular function, nociception and the effects of and motivation to consuming alcohol [89-95], which will be discussed later on.

With regard to stressful stimuli, this can activate the EW Ucn1 system, which is apparent by increased expression of Fos protein in the Ucn1 neurons or increased Ucn1 mRNA [96]. Moreover, with chronic stress, this leads to increased expression of Fos but a habituation of Ucn1 mRNA transcription [97]. In a different manner, however, chronic increases in brain CRF activities are seen to reduce the activity of the Ucn1-immunoreactive neurons in the EW, suggesting, in homeostatic or chronic stress responses, an inverse relationship between the CRF and EW Ucn1 systems [91,98].

Comparable to Ucn1, Ucn2 is expressed in the subcortical areas of the brain. The mRNA is confined to the supraoptic nucleus and paraventricular nucleus (PVN) and in several caudal brainstem and caudal spinal cord motor neuron nuclei [99]. Unlike Ucn1, Ucn2 has further expression in the arcuate nucleus of the hypothalamus and the locus coeruleus. Ucn2 has not been studied as much as Ucn1, and thus, the projection targets of its neurons are unknown [68].

Ucn3 has the most anterior distribution of the Ucns with a subcortical expression [67]. The primary sites of Ucn3 synthesis in the forebrain include the median preoptic nucleus of the hypothalamus, a region of the hypothalamus that is bordered by the fornix and the PVN and the dorsal medial amygdala. Ucn3 synthesis can also be detected in the dorsomedial hypothalamus, the magnocellular and parvocellular components of the PVN, a region dorsal to the supraoptic nucleus and the posterior cortical and amygdalohippocampal transition areas of the amygdala. Certain Ucn3-like fibres have an unknown origin, but they project to the ventromedial hypothalamic nucleus and arcuate nucleus. Moreover, Ucn3 neurons from the amygdala project to the ventral premammillary nucleus [100].

Periphery

The Ucns are also ubiquitous in the periphery. Ucn1 expression has been seen in a variety of tissues including adipose tissue, the heart, the gastrointestinal system and immunological tissue and cells [60,101-103]. There is also evidence of the expression of Ucn1-like immunoreactivity with growth hormone expression and, to a lesser extent, prolactin expression. It was only minimally coexpressed with ACTH [104].

Ucn2 gene expression was much more comprehensively detected in the periphery, including the heart, lung, muscle, stomach and adrenal and peripheral blood cells [105,106]. In peripheral rodent tissue, it was apparent that skeletal muscle also contained high levels of Ucn2, amongst a vast range of other tissues [105].

Like Ucn2, *Ucn3* gene expression was also observed in the heart, adipose tissue and skin but at much lower levels [67,101]. *Ucn3* gene expression was also detected in the thyroid, adrenals, pituitary, β-cells of the pancreas, spleen, kidney and muscularis mucosa of the gastrointestinal tract [67,105,107,108].

Physiological Effects of Urocortin

This review will focus on the effects of Ucn in relation to osteoclasts and the bone. However, some of the other general physiological and behavioural effects will be mentioned briefly, and as Ucn is ubiquitous in the body, it is utilised extensively.

Concerning stress response, Ucn1 can activate the pituitary-adrenal axis, leading to the release of ACTH, and glucocorticoids can increase the expression of Ucn2 in the hypothalamus [61,109,110]. Ucns also can facilitate an increase in energy expenditure (by increasing oxygen consumption and sympathetic activity) and a decrease in food intake, via suppressing appetite [62,66,111]. Moreover, Ucn1, mediated by CRF1 receptors, also has anxiogenic-like effects, suggesting a role in anxiety-related conditions [112].

The Ucns, like CRF, are also perceived to have a vasodilatory effect via the arteriole endothelium and cardiac CRF2 receptors, owed to their ability to stimulate various signalling transduction pathways, such as protein kinase A and MAPK [113-115]. This can lead to a reduction in mean arterial pressure. The Ucns can also have a cardioprotective and recovery effect post-myocardial infarction [116,117]. Immunohistochemistry results have also demonstrated that the Ucn peptide is also present in the lungs. Giving Ucn aerosol to rats leads to pulmonary oedema and contributed to an increase in lung vascular permeability [118].

Ucns have an inhibitory effect on gastrointestinal motility by inhibiting gastric emptying and inducing gastric stasis [119]. In addition, in a murine model of Crohn’s disease, Ucn1 has been shown to significantly reduce the severity of weight loss, diarrhoea and inflammatory colitis. Ucn1 can restore mucosal immune tolerance and inhibit the inflammatory response [120].

Ucn2 binding to CRF2 receptors has also shown to play an important role in the regulation of myometrial contractions during pregnancy and labour; this is achieved via modulating myosin light chain phosphorylation [121]. Very recently, Ucn1 (via antagonising CRF2 as opposed to its preferred CRF1 receptor) has exhibited the suppression of the migration of endometrial cancer cells in vivo but has no effect on the proliferation of these cells [122].

In the remit of regenerative medicine in the musculoskeletal system, Ucn has been shown to have potential roles. Ucn1 can curtail the autoimmune response and the inflammatory response in rheumatoid arthritis, which are the two main factors of the disease [56]. In another inflammatory disease, osteoarthritis, it was observed that the Ucn1 plays a vital role in chondrocyte survival, and Ucn1 expression was increased when apoptotic stimuli presented, such as NO [57].

Urocortin 1 in osteoporosis

Osteoclast Maturation

Osteoporosis is not considered to be an inflammatory disease like rheumatoid arthritis or osteoarthritis. However, a study conducted by Combs et al. [123] in 2012 demonstrated that Ucn1 has a role in inhibiting processes that contribute to an increase in osteoclast resorptive activity.

Osteoclasts mature into large multinucleated tartrate-resistant acid phosphatase (TRAP) positive cells [58]. Combs et al. [123] observed that Ucn1 inhibits this process, as evidenced by a significant reduction of TRAP-expressing osteoclasts. It was also found that there was downregulation of the expression of several osteoclast markers, such as calcitonin receptor (CT-R), cathepsin K (CatK), TRAP and dendritic cell-specific transmembrane protein (DC-STAMP) [123]. The expression of these markers illustrate osteoclast maturation, as they are mainly expressed by osteoclasts and not by macrophages [124]. These processes thus indicate that Ucn1 was inhibiting maturation.

Osteoclast Function

Ucn1 may also inhibit osteoclastic bone resorption. In this study, incubation with a half maximal inhibitory concentration (IC_50_) value of 10^-9^ M of Ucn1 leads to a significant decrease in bone resorption, and a value of 10-7 M leads to a complete lack of bone resorption. This was demonstrated by the significant reduction of actin rings and the total resorption pit areas in bone slices, which were seen in control samples. It is believed that this was facilitated via closing transient receptor potential channel 1 (TRPC1)-like cation channels, which were located on the osteoclasts [123]. The TRPC1-like cation channel is involved in mediating calcium entry into the plasma membrane to counter reduced endoplasmic calcium stores [125]. In addition, a study revealed that oestrogen-deficient mice, which underwent ovariectomies and TRPC1 knockout, were seen to have higher trabecular bone density in comparison to wildtype mice and typical TRPC1 knockout mice [126]. This suggests TRPC1 having a role in osteoporosis, which are closed by Ucn1, subsequently inhibiting bone resorption.

Moreover, as mentioned earlier, osteoclasts form actin-rich structures that are involved in the bone resorptive process via creating a ‘sealing zone’. After the incubation of Ucn1 with an IC_50_ value of approximately 5×10^-5^ M, actin ring formation was significantly reduced, and at 10^-7^ M, there was a complete lack of any actin ring structures. It should be further noted that Ucn1 did not affect osteoclast apoptosis at all, judging by caspase 3 and poly (ADP-ribose) polymerase (PARP) cleavage, which are both indicators of the final stages of apoptosis [123].

Osteoclast Motility

Combs et al. [123] also investigated the effect of Ucn1 in osteoclast motility. Pseudopodial ruffling suggests osteoclast motility, and this ruffling was reduced significantly within 30 minutes after the administration of Ucn at 10^-7^ M incubation. This was accompanied with the arrest of cytoplasmic motility [123]. However, the underpinned mechanisms are not yet known.

Urocortin System Distribution

Combs et al. [123] discovered that osteoclasts express the CRF2(β) receptor subtype. Pre-osteoclasts expressed the CRF2(β) receptor subtype also but did not express Ucn1. Osteoblasts, on the other hand, expressed CRF-BP, whereas osteoclasts did not express this binding protein. It is very much possible that Ucn1 may serve as an autocrine regulator of osteoclast function and as a paracrine regulator of osteoclast maturation. CRF-BP expression by osteoblasts could influence Ucn1 concentration [123].

On the contrary, regarding the type 2 Ucns, Ucn2 and Ucn3, their presence in osteoclasts and osteoblasts is currently unknown. It is possible that they may influence one, if not more of, maturation, function or motility of these cells [123].

Signalling Pathway in Bone Tissues

Considering there is a large variety of signalling pathways the Ucn ligands use, the exact downstream signalling pathway that lends Ucns their anti-resorptive properties has not yet been identified. The G alpha s (Gαs) and G alpha q (Gαq) pathways have been considered to fulfil this role. This is suggested due to their association with the effects Ucn1 enacts in other tissues, such as neuronal cytoskeletal restructuring. It was demonstrated that CRF (which preferentially binds to CRF1 receptors) can trigger neuronal cytoskeletal restructuring via the Gαs signalling pathway; on the other hand, Ucn2 (which only binds to the CRF2 receptor with high affinity) inhibits neuronal cytoskeletal restructuring via the Gαq signalling pathway [127]. With this in mind, the Gαs and Gαq pathways are not specific to the CRF1 and CRF2 receptors, respectively. It is therefore still unknown which pathway lends the Ucns their anti-osteoporotic characteristics.

Future perspectives of urocortin

Urocortin has demonstrated anti-resorptive properties in mice osteoclasts and shows promise for being developed as a therapy. In a project in 2015-2016, the host laboratory has also investigated the Ucn system (primarily Ucn1) and its role in osteoclasts in rodents. They confirmed that Ucn1 inhibits osteoclast resorption and motility and modulates the actin cytoskeleton.

It is very reasonable to expect that Ucn can be developed as a therapy for osteoporosis, given its potential with other degenerative musculoskeletal diseases. With regard to rheumatoid arthritis, Ucn treatment targeted and reduced the two main components of the disease: autoimmune response and inflammatory response [56]. In osteoarthritis, where chondrocyte apoptosis is one of the major components of the disease, Ucn has displayed chondroprotectivity against proapoptotic stimuli, such as NO [57].

CRF receptors are found throughout the body; thus, a fundamental problem with Ucn as a therapy is its incredibly vast array of effects on various body systems aside from the bone. This introduces the possibility of substantial intolerable side effects. Having said that, there is promiscuity of Ucn signalling; different signalling mechanisms are triggered by the various CRF receptors upon binding with specific Ucn paralogs.

Therefore, there are unanswered questions with regard to the efficacy of Ucn in human tissues (as opposed to rodents) and also the signalling pathway that Ucn exploits for its anti-resorptive traits. It has been hypothesised by the host laboratory that Ucn1 acts through the Gαq pathway and not the Gαs pathway but requires confirmation in human osteoclasts. There are some differences between the murine and human Ucn/CRF/CRF-BP axis; for example, there are different binding affinities.

However, once these questions have been answered, it may be possible to isolate the aforementioned signalling pathway that could then be utilised as a therapy. Such a therapy would have the potential to cease the progression of osteoporosis and would be welcomed greatly with millions of sufferers of this condition.

## Conclusions

Osteoporosis is one of the most serious and widespread degenerative diseases in the world. Particularly in developed countries, human longevity sees improvements each year, and thus, the incidence of osteoporosis will increase. Over time, osteoblasts and their respective growth factors lose their potency, and in postmenopausal osteoporosis, the deficiencies in oestrogen lead to increased bone resorption by osteoclasts. Insufficient dietary calcium, particularly at a young age, can lead to a stunted peak bone mass, which can set one up for age-related osteoporosis in old age. To prevent debilitating and often life-threatening fractures, there are various measures one can take to try and reduce the risk of fractures or to slow the progression of osteoporosis. However, they often are accompanied by side effects of varying severity, and none of these therapies are completely curative.

The anti-resorptive molecule, urocortin, is believed to have a potential role in regulating bone remodelling and thus osteoporosis. The urocortin system, notably involving Ucn1, works against the RANKL/RANK/OPG system, where it has been seen to inhibit the maturation, function and motility of osteoclasts. This will conceivably reduce the severity of osteoporosis and the risk of fractures. As of now, there are still unanswered questions that need to be resolved. There is no evidence that Ucn1 inhibits human osteoclasts nor Ucn2 and Ucn3 having any anti-osteoporotic effect. In addition, the exact signalling pathway utilised by the urocortin system axis to achieve its anti-resorptive effects is unknown. If Ucn1 itself cannot be used as a drug due to the ubiquitous presence of its receptors in the body, part of the relevant downstream pathway can be targeted and used as a therapy base. The use of urocortin is an exciting prospect, which will hopefully continue to be fully exploited so that it can be translated into clinical practice and revolutionise osteoporosis treatment.
